# TGF-β and NF-κB signaling pathway crosstalk potentiates corneal epithelial senescence through an RNA stress response

**DOI:** 10.18632/aging.101050

**Published:** 2016-10-06

**Authors:** Zhi-Yuan Li, Zhao-Li Chen, Ting Zhang, Chao Wei, Wei-Yun Shi

**Affiliations:** ^1^ State Key Laboratory Cultivation Base, Shandong Provincial Key Laboratory of Ophthalmology, Shandong Eye Institute, Shandong Academy of Medical Sciences, Qingdao, China; ^2^ Qingdao University Medical College, Qingdao, China

**Keywords:** corneal epithelium, cellular senescence, transforming growth factor-β, RNA stress granules, nuclear factor-κB

## Abstract

The corneal epithelium plays important roles in the maintenance of corneal transparency for good vision, and acts as a protective barrier against foreign insults. Structural and functional changes with aging in the corneal epithelium have been documented. Here we found that transforming growth factor-β (TGF-β) is highly expressed in the elderly donor corneal epithelium, as are senescence-associated genes, such as p16 and p21. In human corneal epithelial cell (HCEC) models, TGF-β induces cellular senescence, characterized by increased SA-β-gal positive cells and elevated expression of p16 and p21. Pharmacological inhibition of TGF-β signaling alleviates TGF-β-induced cellular senescence. In addition, we determined that senescence-associated inflammation was significantly aggravated in TGF-β-induced cellular senescence by detecting the expression of interleukin-6 (IL-6), IL-8, and tumor necrosis factor alpha (TNFα). Both genetic and pharmacological approaches revealed that blocking nuclear factor-κB (NF-κB) signaling not only inhibited the production of inflammatory factors, but also rescued the senescent phenotype induced by TGF-β in HCECs. Mechanistically, TGF-β induced an atypical RNA stress responses, leading to accelerated mRNA degradation of IκBα, an inhibitor of NF-κB. Together, our data indicate that TGF-β-driven NF-κB activation contributes to corneal epithelial senescence via RNA metabolism and the inflammation blockade can attenuate TGF-β-induced senescence.

## INTRODUCTION

The corneal epithelium is the outermost layer of the cornea that is in direct contact with the outside environment, such as UV radiation and opportunistic and pathogenic organisms. It is a stratified structure featuring a 5–7-cell-layer non-keratinized squamous epithelium that is essential for corneal clarity and thus for vision. Functionally, it is an integral component of the ocular defense system, providing a protective barrier for the cornea against pathogen invasion. Likewise, it plays an important regulatory function in the passage of solutes and macromolecules [1]. The homeostasis and self-renewal of the corneal epithelium, like many other epithelial tissues, is tightly controlled. However, ocular diseases that compromise the epithelium, including corneal hypesthesia, diabetic keratopathy, limbal stem cell deficiency, dry eye (DE) disease, exposure keratopathy, and neurotropic keratopathy, can disrupt homeostatic turnover of the corneal epithelium and ultimately result in devastating consequences to vision and to the cornea's barrier function. Thus, a full understanding of the biological characteristics of the corneal epithelium affords great insight into the health and disease of the ocular surface.

With age, structural and functional changes can be observed in the corneal epithelium. Although central corneal epithelium thickness seems to remain constant, the paracentral corneal epithelium, as well as the nasal and temporal limbal epithelium, becomes thinner over time [2]. The surface of the superficial cells of the corneal epithelium become smoother with advancing age due to the loss of microvilli, microplicae, and glycocalyx [3]. The basal epithelial cell density in the limbus-corneal and limbus-palisade regions also decreases significantly with age [4]. Besides the structural alterations, functional changes can also be reported. Aging of the ocular surface not only delays corneal wound-healing after laser epithelial keratomileusis (LASEK) [5], but also causes major eye diseases and results in substantial costs in medical and social terms, including DE [6]. Although the age-related senescent phenotype in the normal corneal epithelium has been depicted, the mechanism behind it has not been explicitly illustrated.

Cellular senescence is characterized by irreversible proliferative arrest and a series of morphological and functional alterations, and may further contribute to the decline in organ function and to reduced or lost vitality in organisms [7–9]. Moreover, through the acquisition of a senescence-associated secretory phenotype (SASP), senescent cells can reinforce senescence and activate immune surveillance, and paradoxically have pro-tumorigenic properties [10–12]. Cellular senescence can be induced by various stimuli and stress, including oxidative stress, DNA damage, metabolic insults, oncogene activation, and epigenetic changes [13–17]. Cumulative findings suggest that transforming growth factor-β (TGF-β) is also capable of inducing cellular senescence [17–21]. For instance, TGF-β has been shown to induce both replicative and premature senescence in epithelial cells and to suppress hTERT expression via a Smad3-mediated signaling pathway [22]. Increased levels of TGF-β have been reported in several ocular surface diseases, and have been implicated in their pathogenesis, including pterygium, vernal keratoconjunctivitis (VKC), atopic keratoconjunctivitis (AKC), graft-versus-host disease, and in the DE conjunctiva [23]. However, whether increased levels of TGF-β occur in the corneal epithelium during aging, and involve epithelial senescence, is still unknown.

In the present study, we investigated the role of TGF-β in the aging corneal epithelium. We found increased expression of TGF-β and cellular senescence in the corneal epithelium with age. We demonstrated for the first time that TGF-β could induce human corneal epithelial cell (HCEC) senescence and senescence-associated inflammation through RNA stress responses. Inhibition of TGF-β signaling or NF-κB signaling rescued TGF-β-induced senescence and blocked senescence-related inflammation. Our findings provide insights into the possible contribution of TGF-β to age-related ocular epithelial cellular senescence and senescence-related inflammation and, in turn, to age-related ocular diseases, as well as potential therapeutic targets to alleviate age-related ocular diseases.

## RESULTS

### Increased cellular senescence in the corneal epithelium with age

**Figure 1:**
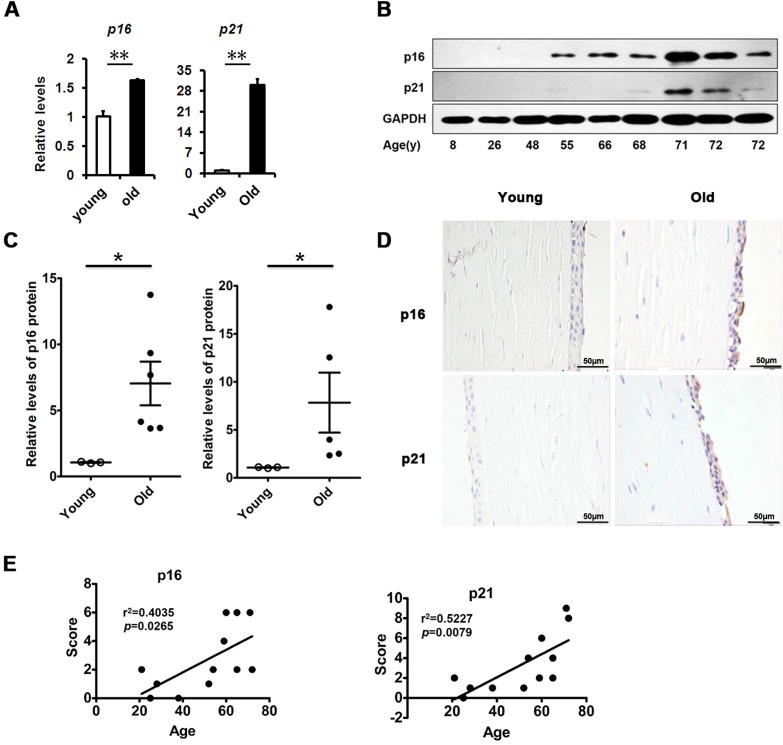
Senescence associated markers rise with age in human corneal epithelium **(A)** The mRNA expression of p16 and p21 in young donors (≤30 years of age) and old donors (≥50 years of age) corneal epithelium(**P≤0.01, n=3).**(B)** Immunoblot analysis of p16 and p21 in the corneal epithelium during aging. **(C)** Quantification of western blots processed with Image J. **(D-E)** Representative photographs **(D)** and histopathology scores **(E)** for the IHC staining of p16 and p21 in corneal epithelium from donors of different ages. There were statistically significant differences in p16 and p21 expression between the donors of younger than 30 years and older than 50years of age (P≤0.05). The figure depicts a Pearson correlation of p16 and p21 protein expression with age **(E)**.

Although age-related structural and functional changes in the corneal epithelium have been documented, the mechanisms underlying them are still unknown. We inferred that corneal epithelial senescence may be a substantial part of the changes. Therefore, we tested cellular senescence in the corneal epithelium. We found that the levels of senescence-related genes in the elderly corneal epithelium, such as *p16* and *p21*, were much higher than in the young corneal epithelium (Fig. 1A). Similarly, the protein levels of them in the elderly corneal epithelium were also elevated, as presented by western blot (Fig. 1B, C). Interestingly, we further found that p16 and p21 protein expressions increased in an age-dependent manner (r^2^=0.4035 and 0.5227, respectively), while elevated expressions of p16 and p21 were seen in elderly corneal epithelium (Fig. 1D, E). Our results suggest that increased cellular senescence occurs in the corneal epithelium with age.

### Elevated TGF-β in the corneal epithelium with age

**Figure 2:**
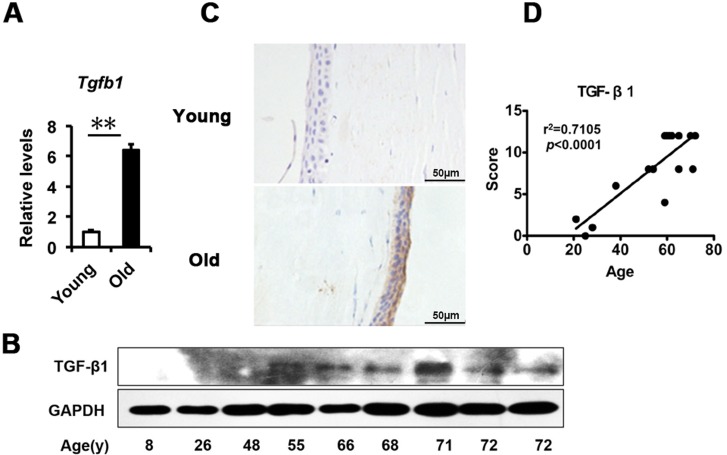
TGF-β1 excess in old donor corneal epithelium **(A)** The mRNA expression of *Tgfb1* in young donors and old donors corneal epithelium(**P≤0.01, n=3). **(B)** Immunoblot analysis of TGF-β1 in the corneal epithelium during aging. **(C-D)** Representative photographs **(C)** and histopathology scores **(D)** for the IHC staining of TGF-β1 in corneal epithelium from donors of different ages. There was a statistically significant difference in TGF-β1expression between the donors of younger than 30 years and older than 50years of age (P≤0.01). The figure depicts a Pearson correlation of TGF-β1 expression with age **(D)**.

Based on the above findings in the corneal epithelium, we questioned what triggered the senescence. A large body of evidence indicates that TGF-β signaling is involved in age-related ocular disease and in cellular senescence. Therefore, we analyzed the expression of TGF-β in the corneal epithelium. We found that the transcriptional level of *TGF-β1* quantified by real-time PCR was significantly increased in the elderly compared to younger corneal epithelium (Fig. 2A), as well as shown in protein levels using western blot (Fig. 2B). The TGF-β protein level detected with immunohistochemistry was also increased in an age-dependent manner (r^2^=0.7105), with strong staining in older corneal epithelium, but weak staining in younger ones (Fig. 2C, D). Together with the literature, these results suggest that the increased TGF-β found in the corneal epithelium with aging may correlate to the increased senescence.

### TGF-β induces cellular senescence in HCECs with increased production of inflammatory mediators

**Figure 3:**
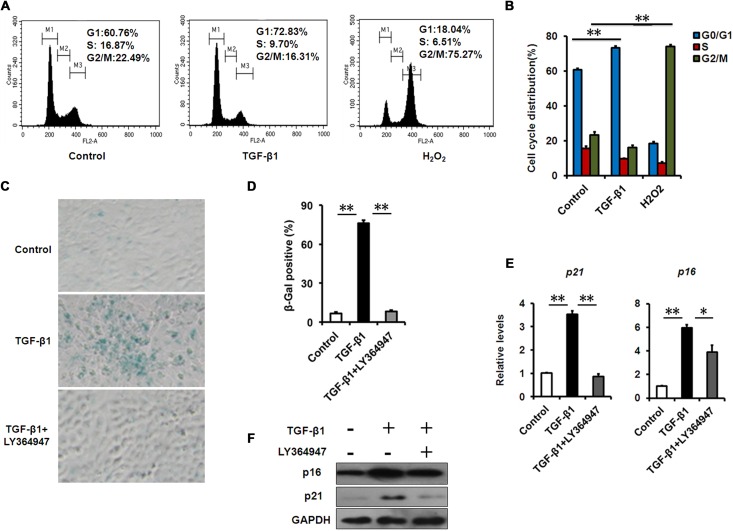
TGF-β1 induces cellular senescence in HCECs **(A-B)** The G1 phase arrest was induced by TGF-β1 treatment. Control and TGF-β1–treated HCECs were subjected to cell cycle analysis after 48h of culture. HCECs treated with H_2_O_2_ (200μM) were taken as positive control. A representative flow cytometric analysis of the DNA content was shown in **(A)** and the values are mean±SD **(B)**. **(C)** HCECs were treated with TGF-β1 (10 ng/ml) alone, or in combination with LY364947 (2μM) for 3days, and tested for SA-β-Gal activity. **(D)** The percentage of SA-β-gal -positive cells in HCECs. **(E-F)** The mRNA **(E)** and protein **(F)** expression of p16 and p21 in HCECs induced by TGF-β1. Bar graphs represent mean±SD. **P≤0.01,*P ≤0.05 vs. control. Data are representative of three independent experiments.

To assess the effect of TGF-β1 on corneal epithelial senescence, we used HCECs as models. Cellular senescence is defined as an irreversible arrest of mitotic cells at the G1 phase, but some cancer cells enter senescence at the G2 or S phase [24]. Cell cycle analysis by flow cytometer showed that the HCECs accumulated at G1 phase (from 60.76% to 72.83%) with a concomitant depletion of S phase cells (from 16.87% to 9.70%) after TGF-β1 exposure (Fig. 3A, B), suggesting that cell cycle arrest during HCECs senescence induced by TGF-β1 occurred at G1 phase, while H_2_O_2_ induced an obvious G2/M phase arrest. In association with the G1 arrest, we also found TGF-β1 increased the percentage of SA-β-gal–staining cells (Fig. 3C, D) and concomitantly increased the expression of p16 and p21, as analyzed by real-time PCR or western blot (Fig. 3E, F). In order to further confirm the effect of TGF-β on cellular senescence, we interrupted the TGF-β signaling pathway using a specific inhibitor, LY364947. When treated with LY364947, the percentage of SA-β-gal–staining cells was significantly decreased (Fig. 3C, D), and the levels of p21 and p16 were also downregulated (Fig. 3E, F). Taken together, these findings suggested that TGF-β1 was able to induce senescence in HCECs.

**Figure 4:**
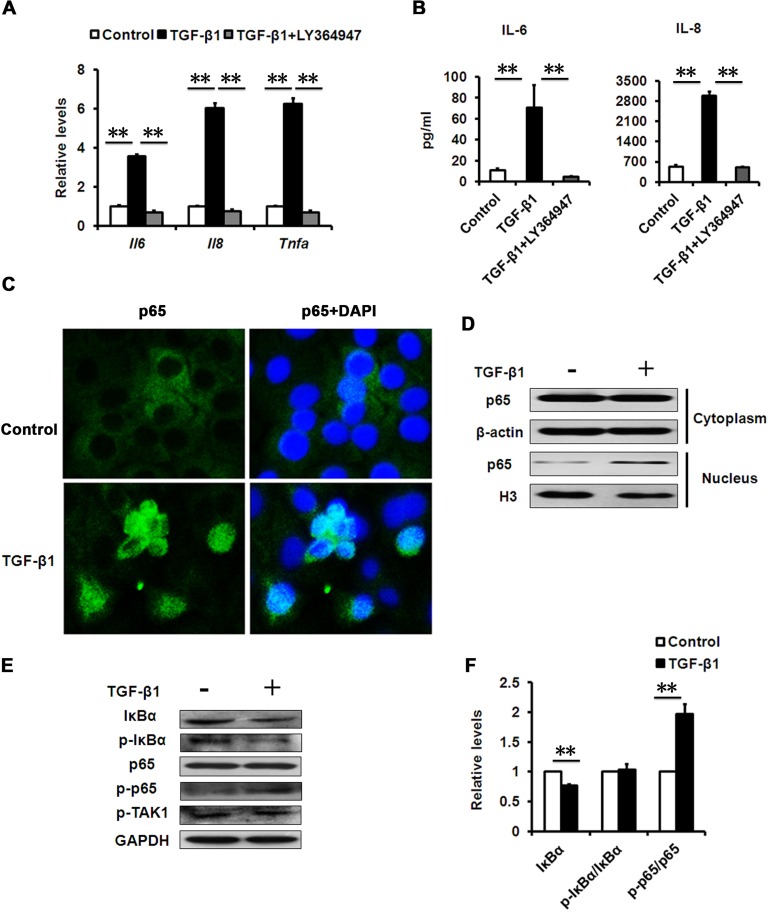
Effects of TGF-β1 on SASP production **(A)** mRNA levels of the indicated genes in HCECs treated with TGF-β1 (10 ng/ml) for 3days. **(B)** The IL-6 and IL-8 in cultured HCECs supernatants were detected by ELISA. **(C)** HCECs were stimulated with TGF-β1 (10 ng/ml) for 24 h. NF-kB p65 translocation was detected by fluorescent microscope. **(D)** HCECs were treated with TGF-β1 (10 ng/ml) for 24 h. Cytoplasmic and nuclear levels of NF-kB p65 were analyzed by Western blot. **(E)** Western blots of whole cell extraction from HCECs treated with TGF-β1 (10 ng/ml) for 24h. **(F)** Quantification of western blots processed with Image J. **P≤0.01. Data are representative of three independent experiments.

It is well-known that aged and senescent cells develop a complex SASP [25]. Further evidence demonstrates that increased production of inflammatory mediators, such as interleukin (IL)-6 and -8, during aging play a substantial role in the establishment and maintenance of the senescent phenotype [12, 26]. To test whether senescence-associated inflammation happens during TGF-β1-induced cellular senescence in HCECs, we measured the expression levels of the key inflammatory mediators during cellular senescence. In TGF-β-induced senescent cells, elevated IL-6 and IL-8 gene expression (Fig. 4A) and secretion (Fig. 4B) was induced, as well as tumor necrosis factor alpha (TNFα) (Fig. 4A). More importantly, LY-364947 treatment not only rescued cells from TGF-β-induced cellular senescence, but also inhibited the expression of IL-6 and IL-8, indicating diminished senescence-associated inflammation (Fig. 4A, B). Numerous studies show that the expression of inflammatory factors, such as IL-6, IL-8 and TNFα, mainly relies on NF-κB signaling pathway. We found that NF-κB signaling was significantly activated in TGF-β-induced senescent HCECs, as evaluated by nuclear translocation of p65 using immunofluorescence and western blot (Fig. 4C, D). These results demonstrated that TGF-β signaling controls the expression of inflammatory mediators through NF-κB signaling during cellular senescence.

### NF-κB activation by TGF-β contributes to senescence-associated inflammation

It has been widely accepted that TGF-β has an anti-inflammatory role in immune responses, but in certain pathological conditions, TGF-β can also be pro-inflammatory [27]. However, its pro-inflammatory roles are still unclear. Because NF-κB has been shown to mediate inflammation in aging [28], we analyzed whether NF-κB signaling components were differentially expressed in senescent HCECs induced by TGF-β1 versus that of pre-senescent cells. Compared with pre-senescent cells, TGF-β1 treatment led to significant reduction in IκBα protein levels (Fig. 4E, F). IκBα is a canonical and specific inhibitor of NF-κB, and its reduction suggested elevated NF-κB activity in TGF-β-induced senescent cells; our subsequent results confirmed this. IκBα loss is the key step that immediately releases cytoplasmic NF-κB for nuclear translocation and the subsequent phosphorylation of p65, a subunit of NF-κB. Therefore, we measured the phosphorylation of p65 in HCECs treated with or without TGF-β. Much more phosphorylation of p65was found in TGF-β-treated HCECs than in untreated cells (Fig. 4E, F). The results indicated that the TGF-β signaling pathway increased the NF-κB activity.

Phosphorylation of IκBα induced by kinase is a crucial reaction for IκBα degradation and NF-κB activation in classic inflammation. However, we noted that the phosphorylated levels of IκBα did not change when normalized by IκBα protein levels (Fig. 4F). We also found that the TGF-β treatment did not change the phosphorylation of TGF-β-activated kinase-1 (TAK-1), which can mediate TGF-β-induced NF-κB activation (Fig. 4E). Altogether, the results suggest that TGF-β probably uses a mechanism through directly targeting IκBα, rather than upstream kinase signaling, to activate NF-κB in HCECs.

### RNA stress response activates NF-κB

**Figure 5:**
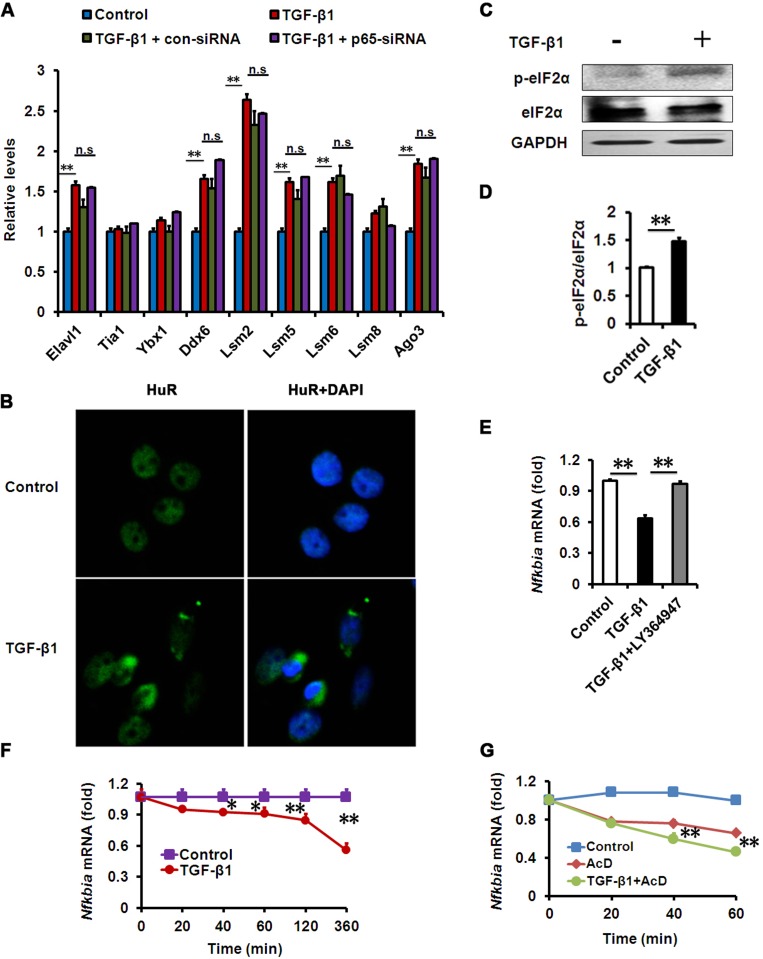
Effects of TGF-β1 on HCECs RNA SGs and IκBα mRNA decay **(A-B)** mRNA levels of SG and PB components **(A)** or HuR immunostaining **(B)** in HCECs treated with TGF-β1 (10 ng/ml) for 24h alone, or in combination with indicated siRNA. Nuclear staining by DAPI revealed cells in sections. Images show representative HuR-containing aggregates. **(C-D)** Phosphorylation of eIF2α was detected by western blot after TGF-β1 (10 ng/ml) treatment for 24h. **(E)** mRNA levels of IκBα in HCECs treated with TGF-β1 (10 ng/ml) alone, or in combination with LY364947 (2μM) for 24h. **(F-G)** IκBα mRNA levels in HCECs treated with TGF-β1 (10 ng/ml) **(F)** or actinomycin D (AcD) alone or AcD together with TGF-β1 **(G)** for the indicated durations. **P≤0.01,*P ≤0.05.

In order to explore how TGF-β could activate NF-κB in a kinase-independent manner, attention was paid to the regulation of mRNA levels. In coping with inflammatory stress, eukaryotic cells can develop a process known as an RNA stress response, characterized by RNA stress granules (SGs) and processing bodies (PBs), to control the degradation of mRNA [29, 30]. In this regard, we speculated that RNA stress induced by TGF-β could be implicated in the degradation of IκBα mRNA. Therefore, we tested whether TGF-β-induced NF-κB activation in HCECs relies on this mechanism. First, we analyzed the expression of these RNA SG and PB genes in our HCEC models, and found that many of them were increased in TGF-β-treated HCECs (Fig. 5A). Consistent with this observation, we examined whether the morphology of RNA SGs could be detected in TGF-β-treated HCECs. We stained HuR, a molecular component of RNA SGs, and found that HuR-positive aggregates presented in TGF-β-treated HCECs but barely presented in untreated cells (Fig. 5B). The phosphorylation of the alpha subunit of eukaryotic translation initiation factor (eIF2α) is essential for SGs assembly [31]. We further measured the phosphorylation levels of eIF2α in HCECs treated with TGF-β, and found that it was much higher after TGF-β1 exposure (Fig. 5C, D). These data suggest that an RNA stress response could be substantial part of the TGF-β-induced inflammation in HCECs.

RNA SGs and PBs can mediate the degradation of mRNA by targeting AU-rich elements (AREs) in the 3′ untranslated region while an ARE is conserved in IκBα mRNA across species [29, 32, 33]. Therefore, we further examined the degradation of IκBα mRNA in TGF-β-treated HCECs. We found that IκBα mRNA levels in HCECs were significantly lower following TGF-β treatment than in the untreated cells, while pharmacologic inhibition of TGF-β signaling could rescue the reduction of IκBα mRNA (Fig. 5E, F). When co-treated with TGF-β and transcriptional inhibitor actinomycin D (AcD), the decay of IκBα mRNA was more potent than AcD treatment alone (Fig. 5G), demonstrating that TGF-β has a strong effect on IκBα mRNA decay. These findings suggest that TGF-β1 induced RNA SG formation and IκBα mRNA degradation, contributing to NF-κB activation in HCECs.

We also tested whether the human donor corneal epithelium displayed evidence of RNA stress and inflammation. The relative levels of RNA SG- and PG-associated genes in elderly donor corneal epithelium were markedly increased compared to young donors (Fig. S1A). Immunofluorescence analysis showed that HuR-positive aggregates presented in elderly donor corneal epithelium, but only barely presented in younger ones (Fig. S1B). IL-8 and IL-6 mRNA abundance was also increased in elderly donor corneal epithelium (Fig. S1C), and immunofluorescence studies corroborated the increased levels of IL-8 in these epithelium (Fig. S1D). Collectively, these data provide evidence of increased RNA stress and aggravated inflammation *in situ* in elderly donor corneal epithelium, mirroring the data for HCECs treated with TGF-β *in vitro*.

### NF-κB inhibition attenuates TGF-β-induced senescence and senescence-associated inflammation

**Figure 6:**
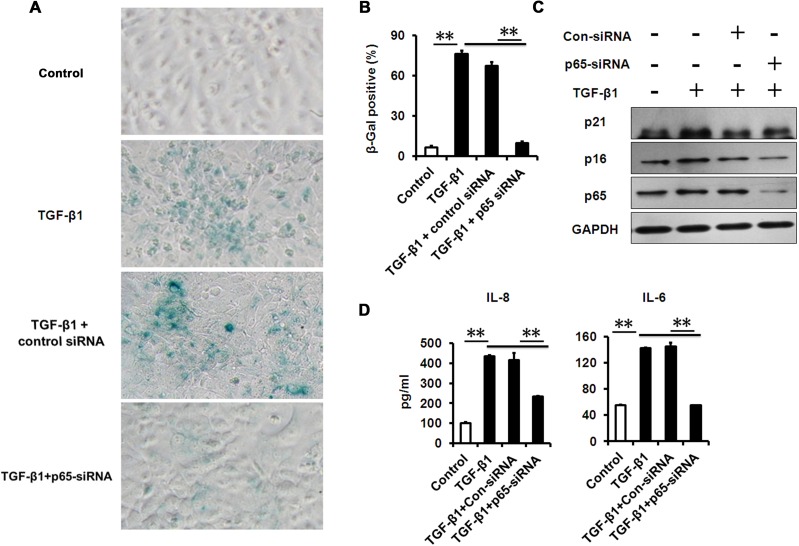
NF-κB p65-konckdown attenuates TGF-β1 induced senescence and SASP **(A-B)** SA-β-Gal activity and the percentage of SA-β-gal-positive cells in HCECs treated with TGF-β1 for 3 days alone, or in combination with indicated siRNA. **(C)** Western blot analysis of p16 and p21 in HCECs treated with TGF-β1 for 3 days alone, or in combination with indicated siRNA. **(D)** The IL-6 and IL-8 in cultured HCECs supernatants were detected by ELISA. **P≤0.01. Data are representative of three independent experiments.

A growing amount of evidence indicates that chronic inflammation accelerates senescence, while inflammation-deletion delays senescence [15, 28, 34]. However, whether NF-κB inhibition could alleviate TGF-β-induced senescence and senescence-associated inflammation in HCECs remains unknown. Therefore, we assessed whether NF-κB inhibition using genetic tools could prevent senescence and senescence-associated inflammation induced by TGF-β. We found that the percentage of SA-β-gal–staining cells in HCECs was notably lower following co-treatment with TGF-β and small interfering RNA specifically targeting p65 (p65-siRNA) versus TGF-β treatment alone (Fig. 6A, B). Furthermore, western blot analysis showed that the expression of p16 and p21 was also significantly decreased in HCECs co-treatment with TGF-β and p65 siRNA than TGF-β treatment alone (Fig. 6C). And as expected, p65 siRNA markedly reduced the protein expression of p65. We also detected the levels of senescence-associated pro-inflammatory mediators, and found decreased IL-8 and IL-6 levels in HCECs co-treated with TGF-β and p65-siRNA than TGF-β alone (Fig. 6D). Similar results were obtained using a chemical inhibitor JSH23, an inhibitor of NF-κB signaling (Fig. S2). In addition, we found p65-siRNA was unable to reverse TGF-β-induced RNA SGs and PBs accumulation (Fig. 5A), indicating that NF-κB was not involved in activating the RNA stress response. Collectively, these results suggest that NF-κB signaling is implicated in TGF-β-induced cellular senescence, and inflammation resolution can rescue HCECs from TGF-β-induced senescence response.

## DISCUSSION

As experimental and clinical evidence has shown, the structure and function of the corneal epithelium are altered with aging. Age-related changes of the corneal epithelium are associated with major eye diseases, including the highly prevalent dry eye disease [6]. It is also known that TGF-β signaling promotes the progression of senescence and stem cell quiescence [35–37]. However, the underlying mechanisms of corneal epithelial changes with aging are largely unknown. Here, we showed that TGF-β1 expression is increased in the elderly donor corneal epithelium, and this age-related expression of TGF-β1 is positively associated with aging of the corneal epithelium. We also found that excess TGF-β1 leads to cellular senescence and NF-κB activation in HCECs in a manner that is engaged in atypical mRNA decay of IκBα.

Our present findings are in line with the literature, showing that increased TGF-β is not only seen in various corneal diseases but is also implicated in their pathogenesis [23, 38, 39]. Otherwise, we should realize that despite this relevance of TGF-β to disease, it also has important biological functions in cell growth, differentiation, and transformation, and the complete deletion of TGF-β is developmentally lethal [40]. With regard to the ocular surface epithelium, a lack of TGF-β signaling can result in epithelial hyperplasia and goblet cell differentiation [41], and can also affect corneal wound-healing [42, 43], suggesting that a normal level of corneal TGF-β is biologically required and thereby protective of the cornea. Therefore, we speculate that increased TGF-β in many ocular diseases may represent an adaptive response. Although increased TGF-β is observed in many ocular diseases, including pterygium, VKC, AKC, and graft-versus-host disease, and in DE conjunctivas, it is unclear how the increased TGF-β affects epithelial cell functionality and contributes to the pathogenic process of such ocular surface diseases. Our results demonstrate that TGF-β expression is increased in the elderly donor corneal epithelium and that excess TGF-β leads to corneal epithelial senescence. Therefore, appropriate TGF-β suppression can present a therapeutic basis for treating aging or age-related ocular diseases. Nevertheless, further studies are necessary to determine whether TGF-β itself or the downstream mechanism represents the most viable target for such treatments.

Senescent cells usually produce a series of cytokines, chemokines, and other soluble factors, a phenotype that is believed to reinforce senescence [12]. NF-κB has been implicated in inflammation in aging and age-related ocular disease. As shown in our study, NF-κB is also required in the senescence induced by TGF-β1. The senescence of HCECs induced by TGF-β1 was markedly attenuated while treated with NF-κB inhibitor JSH23 or p65-siRNA, and in accordance with previous reports, the SASP is dependent on NF-κB [44, 45]. Therefore, some of the SASP factors, such as IL-8, which is also highly expressed in elderly human corneal epithelium, probably contribute to the reinforcement of senescence.

Classical NF-κB activation depends on the IκBα phosphorylation and degradation induced by the activation of kinases, such as IκBα kinase and TAK1 [33]. As a result, activated NF-κB induces an array of gene expressions of inflammatory cytokines. Some researchers have found that TGF-β can induce the production of inflammatory mediators in ocular surface epithelial cells [23]. However, the mechanisms behind this still remain unclear. Here, our results indicated that an RNA stress response induces IκBα mRNA degradation to activate NF-κB in HCECs. It has been reported that the RNA stress response can provide an early intracellular defense during which RNA SGs and PBs are formed to degrade AU-rich elements (AREs)-containing mRNAs [29, 32]. In this study, we demonstrated that IκBα mRNA turnover is fast and is sensitive to RNA granules-mediated mRNA decay in HCECs. Gene sequences analyses showed that AREs are conserved in IκBα mRNAs across species, and that TGF-β can promote IκBα mRNA degradation in the hypothalamus of mice or in mouse hypothalamic GT1-7 cells, which further corroborated our conclusions [33]. Although excess TGF-β can trigger the RNA stress response in HCECs, it remains to be studied whether other contributing factors exist, and how excess TGF-β triggers this process. Furthermore, we found that the induction of NF-κB-triggered inflammatory genes was suppressed by TGF-β signaling inhibition, suggesting that TGF-β is involved in initiating inflammation in HCECs. These findings, coupled with our observation that the human elderly corneal epithelium contains elevated amounts of TGF-β1, IL-6, and IL-8, as well as evidence of increased RNA stress granules, provide a rationale for targeting this pathway in the corneal epithelium. Nevertheless, it remains necessary to further elucidate the details of the associations between TGF-β, NF-κB, and corneal epithelial senescence.

The present findings also reveal a new role for TGF-β signaling in the ocular surface epithelium. TGF-β signaling can be involved in cancer, and can serve as both a tumor suppressor and a tumor promoter. The limbal transition zone is highly susceptible to tumor formation in humans [46], but a link to TGF-β signaling has not been established. The goblet cell expansion and conjunctival epithelial hyperplasia observed in *Tgfbr2*cKO mice does not have the morphological features indicative of a malignant lesion, and maintains an intact basement membrane, indicating that the loss of TGF-β signaling in the ocular surface epithelium is sufficient to promote epithelial hyperplasia but is insufficient for progression to carcinoma. This further suggests that additional insults are required to drive carcinogenesis in the ocular surface [41]. It has been reported that cellular senescence is crucial for tumor suppression and that the SASP reinforces senescence, activates immune surveillance to eliminate premalignant cells, and paradoxically also has pro-tumorigenic properties [47]. The findings in the present study showed that excess TGF-β1 in the ocular surface induces cellular senescence and senescence-associated inflammation, suggesting that cellular senescence induced by increased TGF-β with aging or in age-related ocular disease may be important for anti-tumorigenesis, in addition to its roles in regulating proliferation and differentiation.

In summary, our study shows that excess TGF-β in the aged corneal epithelium can induce cellular senescence and senescence-associated inflammation via an RNA stress response. Our findings enrich the understanding of how age-related diseases of the ocular surface occur with age, thereby providing potential therapeutic targets for age-related ocular diseases.

## MATERIALS AND METHODS

### Human cornea tissue

This study was approved by the Shandong Eye Institute Review Board. The handling of donor tissues was consistent to the tenets of the Declaration of Helsinki of 1975 and its 1983 revision in protecting donor confidentiality. The human donor corneas were provided by the International Federation of Eye Banks, Eye Bank of Shandong (Qingdao, China) and preserved in DX intermediate-term medium at 4°C [48]. Most of the donors had causes that did not comprise corneal or eye disease. The death-to-preservation interval was less than 30 h. The donor corneal rims (residual tissues) used for this study were collected via penetrating keratoplasty (PKP). The corneal rims were divided into several parts for immunofluorescence / immunohistochemistry staining or RNA analysis, respectively. Information of donors with different ages is listed in Table S1.

### Cell culture and treatment

Immortalized HCECs derived from human corneal epithelium were presented by professor Chonn-Ki Joo of the Catholic University of Korea, Seoul, Korea. HCECs were maintained in DMEM/F12 (Gibco, Grand Island, NY) with 10% FBS and penicillin-streptomycin at 37°C in a humidified atmosphere containing 5% CO2. Cells were fasted in serum-free medium for an overnight period and then the medium was replaced by fresh serum-free DF/12 supplemented with 10ng/ml recombinant human TGF-β1 (Peprotech) alone, or in combination with LY364947 (an potent inhibitor of TGFβR, 2μM, Selleck) or JSH-23 (an inhibitor of NF-κB transcriptional activity, 15μM, Selleck).

### siRNA treatment

HCECs which grew to 60-80% confluentin a 6-well plate for about 24 hours after seeding, were treated with both p65 siRNA and control siRNA (Santa Cruz), as previously reported [49]. Briefly, each siRNA treatments were gently mixed with each concentration with siRNA transfection medium and reagent solution at room temperature for 30 minutes, and then overlay the complex solution onto the washed HCECs. After 5-7 hours incubation, add fresh serum-free medium and incubate cells for an additional 18 hours. TGF-β1 treatment was carried out subsequently.

### Immunohistochemistry and Immunofluorescence staining

Immunohistochemical staining and immunofluorescence staining were performed as previously described [50]. For paraffin sections, the residual corneal rims from PKP were immersed in 4% paraformaldehyde (PFA) at 4°C until process. Corneal rims were processed and embedded in paraffin wax. Four-micrometer-thick corneal sections were deparaffinized and antigen was retrieved using a steamer in epitope retrieval solution (Maixin Biotech, Fujian, China). Anti-TGF-β1 (sc146, Santa Cruz), anti-p16 (ab51243, Abcam), anti-p21 (ab109520, Abcam), anti-IL-8 (sc73321, Santa Cruz) and anti-HuR (sc5261, Santa Cruz) were used as primary antibodies and subsequently reacted with secondary antibodies for immunohistochemistry or immunofluorescence staining. Naive IgGs of appropriate species were used as negative controls. For pathological scoring, five fields per sample were examined and scored as described previously to facilitate comparisons [51].

For immunofluorescence staining of HCECs, HCECs grown on 24-well culture plates were fixed in 4% PFA for 15min, penetrated with 0.1% Triton-X 100 for 30min, blocked with 5%BSA for 1h at room temperature and treated with anti-HuR antibody (sc5261, Santa Cruz) or anti-p65 antibody (#4764, Cell Signaling) at 4°C overnight, and subsequently reacted with fluorescent secondary antibody (Abcam), respectively. DAPI staining was used to reveal all cells in the section. Images were taken using a confocal microscope.

### Flow Cytometric Analysis

Cell cycle status in the HCECs was determined by measuring nuclear DNA content. The cells were collected 48hours after treatment with TGF-β1, centrifuged at 300g for 5 minutes, and washed twice with ice-cold PBS. The cells then were fixed in 70% ethanol at 4°C for more than 4 hours. The pellet was collected by centrifugation before the RNase A solution was added (final concentration 10 mg/mL). After a 1-hour incubation at 37°C, the cells were stained with propidiumiodide (PI; final concentration 100 μg/mL) at 37°C for 30minutes. The samples were analyzed using a FACSCalibur flow cytometer (BD Bioscience).

### Quantitative RT-PCR

Total RNA was extracted from the cornea epithelium or cultured HCECs using TRIzol reagent (Invitrogen, Carlsbad, CA, USA) and was reverse-transcribed using reverse transcriptase (TOYOBO, Osaka, Japan). Quantitative real-time PCR analysis was performed on an ABI prism 7500 (Applied Biosystems, Foster City, CA, USA) using SYBR GREEN mix (TOYOBO, Osaka, Japan). Primers used for quantitative RT-PCR were listed in the Table S2.

### Western Blot

For whole cell extraction, the cornea epithelium from residual donor tissues or HCECs treated with TGF-β1 for the indicated time points were lysed in RIPA buffer (P0013B, Beyotime, Beijing, China) with protease inhibitor cocktail (Millipore). After centrifugation (4°C, 10 min, 12,000 g), samples were prepared for Western blot analysis.

For preparation of cytoplasmic and nuclear fraction, HCECs were treated with TGF-β1 for 24h. Nuclear and cytoplasmic proteins of HCECs were extracted using the Nuclear and Cytoplasmic Protein Extraction Kit (P0028, Beyotime, Beijing, China) according to manufacturer's instructions.

Western blot were performed as described previously [52]. The membranes were probed with the following primary antibodies: anti-GAPDH (KC-5G5, Kangchen), anti-β-actin (LK9001, Sungene Biotech), anti-Histone H3 (ab1791, Abcam), anti-p16 (ab51243, Abcam), anti-p53 (ab26, Abcam), anti-p21 (ab109520, Abcam), anti-TGF-β1 (sc146, Santa Cruz), anti-IκBα (sc847, Santa Cruz), anti-p-IκBα (#2859, Cell Signaling), anti-p65 (#4764, Cell Signaling), anti-p-p65 (#3039, Cell Signaling), anti-p-TAK1 (#4531, Cell Signaling), anti-eIF2α(sc133132, Santa Cruz), anti-p-eIF2α (sc293100, Santa Cruz) and subsequently reacted with HRP-conjugated secondary antibodies, respectively (Pierce, 1:3,000). Quantification of western blots was processed with Image J.

### SA-β-Gal staining

HCECs were previously treated with TGF-β1 or indicated chemicals for 3 days. SA-β-gal activity was evaluated using the SA-β-gal Staining Kit (RG0039, Beyotime, Beijing, China) following the manufacturer's instructions.

### ELISA

Levels of IL-6 and IL-8 in cell culture supernatants were measured using commercially available enzyme-linked immunosorbent assay (ELISA) kits (Joyeebio, Shanghai, China).

### Statistical analyses

All experiments were performed at least three different experiments, and data was presented as mean±SD. Two-tailed Student's t-test was used for comparisons between two groups, and one-way analysis of variance (ANOVA) was used for comparisons among more than two groups. A value of p<0.05 was considered statistically significant.

## SUPPLEMENTARY MATERIALS TABLES AND FIGURES


