# Correction for: TGF-β and NF-κB signaling pathway crosstalk potentiates corneal epithelial senescence through an RNA stress response

**DOI:** 10.18632/aging.203516

**Published:** 2021-08-31

**Authors:** Zhi-Yuan Li, Zhao-Li Chen, Ting Zhang, Chao Wei, Wei-Yun Shi

**Affiliations:** 1 State Key Laboratory Cultivation Base, Shandong Provincial Key Laboratory of Ophthalmology, Shandong Eye Institute, Shandong Academy of Medical Sciences, Qingdao, China; 2 Qingdao University Medical College, Qingdao, China

**Keywords:** correction

Original article: Aging. 2016; 8:2337–2354.  . https://doi.org/10.18632/aging.101050

**This article has been corrected:** The authors replaced SA-β-Gal staining panels for Control and TGFβ1 in **Figure 6A**. Originally the same Control image was used in Figure 6A and Supplementary Figure S2A, and the same TGFβ1 image was used in Figure 6A and Figure 3C. The replacement was done with the representative images from the original set of experiments. This alteration does not affect the results or conclusions of this work.

New **Figure 6** is presented below.

**Figure 6:**
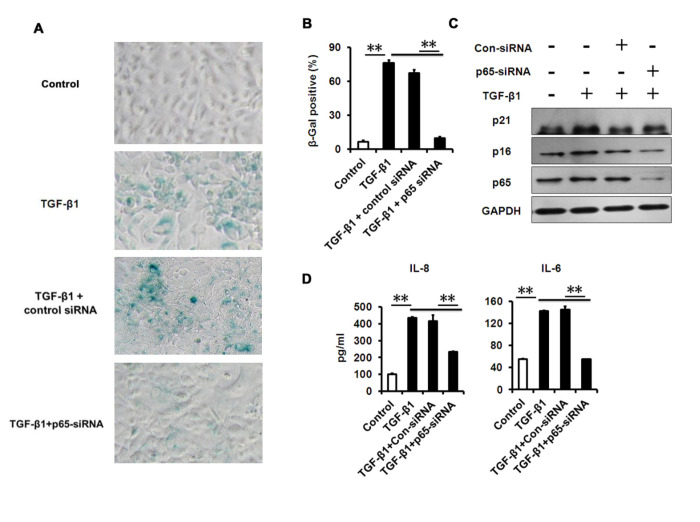
**NF-κB p65-konckdown attenuates TGF-β1 induced senescence and SASP. (A-B)** SA-β-Gal activity and the percentage of SA-β-gal-positive cells in HCECs treated with TGF-β1 for 3 days alone, or in combination with indicated siRNA. **(C)** Western blot analysis of p16 and p21 in HCECs treated with TGF-β1 for 3 days alone, or in combination with indicated siRNA. **(D)** The IL-6 and IL-8 in cultured HCECs supernatants were detected by ELISA. **P≤0.01. Data are representative of three independent experiments.

