# Supplementary material for: TGF-β and NF-κB signaling pathway crosstalk potentiates corneal epithelial senescence through an RNA stress response
Source: Aging (Albany NY). 2016 Oct 6;8(10):2337–50. doi: 10.18632/aging.101050 (PMC5115892; doi:10.18632/aging.101050)
Supplement: Supplementary file 1 [file aging-08-2337-s001.pdf]

SUPPLEMENTARY MATERIAL

Supplemental Table S1. Donor information

Groups	Donor age (Y)
age≦30y	8
	21
	25
30y< age< 50y	26
	28
	38
age≧50y	42
	45
	48
	52
	54
	55
	59
	59
	60
	60
	62
	65
	65

Supplemental Table S2. Primers used for real time-PCR

Gene name	Orientation	Primer sequence (5' - 3')
GAPDH	forward	AGGGCTGCTTTTAACTCTGGT
	reverse	CCCCACTTGATTTTGGAGGGA
P16	forward	CACGGGTCGGGTGAGAGT
	reverse	CCCAACGCACCGAATAGTTAC
P21	forward	GCCTGGACTGTTTTCTCTCG
	reverse	ATTCAGCATTGTGGGAGGAG
P53	forward	TTGAGACTGGGTCTCGCTTT
	reverse	AAATGCAGATGTGCTTGCAG
TGF-β1	forward	CCCAGCATCTGCAAAGCTC
	reverse	GTCAATGTACAGCTGCCGCA
IL-6	forward	GATGAGTACAAAAGTCCTGATCCA
	reverse	CTGCAGCCACTGGTTCTGT
IL-8	forward	ACCGGAAGGAACCATCTCACT
	reverse	GGAAGGCTGCCAAGAGAGC
TNF-α	forward	CAGCCTCTTCTCCTTCCTGAT
	reverse	GCCAGAGGGCTGATTAGAGA
Elavl1	forward	GAGGCTCCAGTCAAAAACCA
	reverse	GTTGGCGTCTTTGATCACCT
Tia1	forward	GGGATATGGCTTTGTCTCCTT
	reverse	GCCACCACCCATCTGTTGAATG
Ddx6	forward	GAAATGGCTTATGCCGCAAT
	reverse	GATGACCAAAGCGACCTGATC
Ybx1	forward	AGATGGAGAGACTGTGGAGTTTGA
	reverse	GGACCCCTACGACGTGGATA
Lsm2	forward	AGCATCTGTGGAACCCTCCAT
	reverse	TGGCAGCTGCACGTATCG
Lsm5	forward	TGTTGGTACTCTTCTAGGATTTGATGA
	reverse	CCTCCAGGAACCAGCATTGTTA
Lsm6	forward	CGGACGACCAGTTGTGGTAA
	reverse	CGGATAAATGCATCCCCATACT
Lsm8	forward	CGTCCGCTTTGGAGAACTACA
	reverse	CCCCCTGTGAAGAGCTGAATAC
Ago3	forward	TGGGAGCCGATGTCACTCA
	reverse	AGGCCAAGTCCTGGATGATCT
IκBα	forward	GATCCGCCAGGTGAAGGG
	reverse	GCAATTTCTGGCTGGTTGG

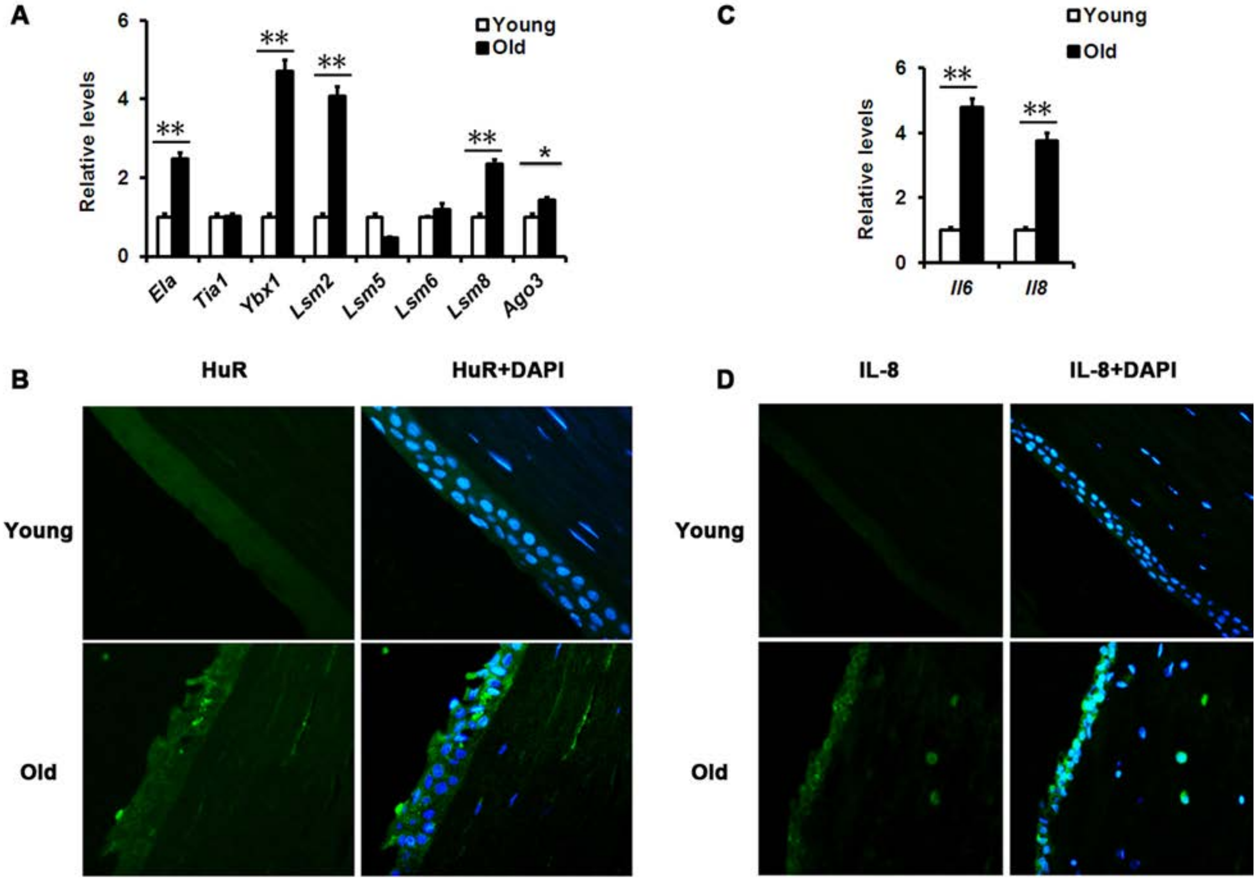

Supplemental Figure S1. RNA stress and SASP in aged human corneal epithelium. (A-B) mRNA levels of SG and PB components **(A)** or HuR immunostaining **(B)** in young donor and old donor corneal epithelium. **(C)** mRNA levels of the indicated SASP genes in young donor and old donor corneal epithelium. **(D)** IL-8 staining in donor corneal epithelium.

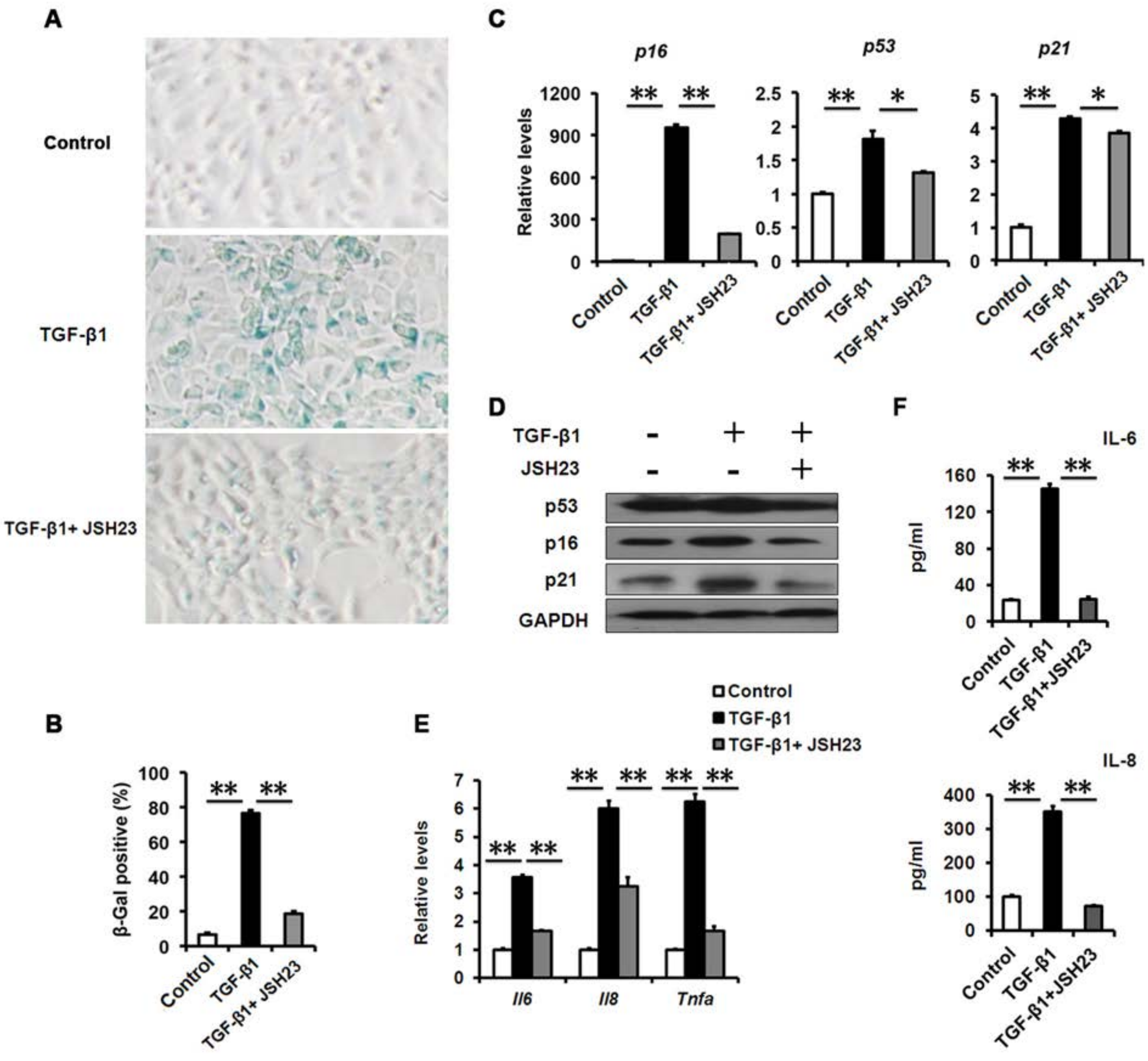

Supplemental figure S2. NF-κB inhibition with JSH23 attenuates TGF-β1 induced senescence and SASP. (A-B) SA-β-Gal activity and the percentage of SA-β-gal-positive cells in HCECs treated with TGF-β1 alone, or in combination with JSH-23 (15µM). **(C-D)** The mRNA and protein levels of p16, p53 and p21 in HCECs treated with TGF-β1 alone, or in combination with JSH-23 (15µM). **(E)** mRNA levels of the indicated SASP genes in HCECs treated with TGF-β1 (10 ng/ml) for 3 days. **(F)** The IL-6 and IL-8 in cultured HCECs supernatants were detected by ELISA.
